# White Matter Abnormalities Based on TBSS and Its Correlation With Impulsivity Behavior of Methamphetamine Addicts

**DOI:** 10.3389/fpsyt.2020.00452

**Published:** 2020-05-21

**Authors:** Sihong Huang, Wenhan Yang, Jing Luo, Cui Yan, Jun Liu

**Affiliations:** Department of Radiology, Second Xiangya Hospital of Central South University, Changsha, China

**Keywords:** methamphetamine, diffusion tensor imaging, tract-based spatial statistics, impulsivity, Barratt Impulsivity Scale

## Abstract

**Background:**

Methamphetamine (MA) abuse is one of the most rapidly growing illicit drug problems worldwide. Impulsivity has been considered as a core impairment underpinning addictive behavior. Studies have demonstrated that MA addicts have white matter abnormalities based on ROIs. There are few studies on whole brain, and the association between whole brain tracts and impulsivity in MA dependence remain unclear. Tract-based spatial statistics (TBSS) was used to detect four DTI measures, and these were correlated with the Barratt Impulsivity Scale (BIS) to verify and expand the previous results.

**Methods:**

A total of 28 MA addicts and 22 healthy controls were recruited. MRI was performed to evaluate the brain structural changes, the BIS was used to evaluate impulsivity behavior, white matter differences were compared between MA addicts and healthy controls, and then determine correlation between diffusion parameters and BIS scores.

**Results:**

MA addicts had significantly lower FA, and higher AD, RD, and MD in a wide range of white matter, which mainly included: corona radiata, internal capsule, superior longitudinal fasciculus, external capsule, inferior fronto-occipital fascicules, posterior thalamic radiation, sagittal stratum, fornix and stria terminalis, cerebral peduncle, superior cerebellar peduncle, corpus callosum, and corticolspinal tract compared with controls. The MA group had significantly higher total score, attention and motor scores compared to healthy controls. Higher MD in the right corticospinal tract was significantly associated with higher total scores.

**Conclusion:**

MA addicts exhibit a globally diminished white matter integrity. furthermore, they present with high levels of impulsivity, and this dysfunction is associated with MD in corticospinal tracts. Future studies on larger sample sizes, gender effects and longitudinal studies are needed.

## Introduction

Methamphetamine (MA) abuse is one of the most rapidly growing illicit drug problems worldwide (1), and studies have suggested that it is second to marijuana in global use (2). In the 2017 World Drug Report, MA was reported to be used by approximately 37 million people all around the world. MA abuse can lead to a variety of diseases, such as HIV, hepatitis B, hepatitis C, and many other diseases spread by blood. Furthermore, MA can alter the release and activity of monoaminergic neurotransmitters, dopamine, norepinephrine, and serotonin (3), and other unknown mechanisms on the central nervous system (CNS), which can be called neurotoxicity. Moreover, studies have shown abnormalities in the structure and function of brains of MA addicts, such as impairments on amygdala (4), and altered cognitive and affective function (5, 6), and even cause abnormalities of their children (7). Hence, this has become a major public health concern, and has caused a big burden to worldwide society.

Diffusion tensor imaging (DTI) is a neuroimaging modality that enables the visualization and characterization of white matter within the CNS by analyzing the three-dimensional diffusion of water molecules within fiber bundles (8). The most widely used measure of water movement is fractional anisotropy (FA), which represents the normalized variance of the total diffusivities (9). Higher FA indicates diffusion that is primarily in a direction parallel to the longitudinal axis of the white matter fiber bundles (9), and this is generally regarded as a marker of more healthy, highly structured, or mature white matter (10). Radial diffusivity (RD) specifically reflects perpendicular diffusion towards membranes. RD is higher when there is less or damaged myelination (11, 12). Axial diffusivity (AD) specifically measures the diffusion along axons, and this has been suggested to be elevated when neurofilaments are damaged (13), or decreased when there is axonal damage (14, 15). Mean diffusivity (MD) represents the global average of diffusion directions, which is higher when there are damaged and/or disorganized white matter tracts (9).

Previous studies have revealed abnormalities in multiple brain regions of MA addicts, including the prefrontal white matter (16–18), striatum (16), corpus callosum (19). In addition, Li Y. et al. reported that the MA group had significantly lower FA in the bilateral basolateral amygdala, subiculum, and nucleus accumbens (20). These impairments are reflected by FA. Another study reported a higher apparent diffusion coefficient (ADC) in the left caudate and bilateral putamen (16). These findings have similarities and differences. In order to clarify and expand these previous findings, the present study measured the FA, MD, RD, and AD. Almost all studies calculated the parameter's value based on the region of interest (ROI). Tract-based spatial statistics (TBSS) can improve the sensitivity, objectivity, and interpretability of analysis of multi-subject diffusion imaging studies *via* carefully tuned non-linear registration, and projecting this onto an alignment-invariant tract representation (21). Few studies have used TBSS. One of them based on ROIs (22). The other based on whole-brain voxels and no differences were found in DTI measurements between the methamphetamine dependence without a history of psychosis and controls (23). In order to verify and expand these previous results, TBSS was used to detect the abnormalities of MA addicts based on the whole-brain voxels in the present study.

Some researches on MA addicts have correlated diffusion parameters with clinical data, as well as with neuropsychological scales: a study revealed the correlation between diffusion parameters and MA use (e.g., the initiation of MA use, daily amounts, and cumulative lifetime dose) (16). Another study revealed correlations between white matter FA and psychiatric symptoms (e.g., depression) (18). The study conducted Kim et al. revealed a significant negative correlation between the Wisconsin Card Sorting Test (WCST) total error and FA in the genu region of the corpus callosum, and illustrated the relationship between white matter integrity and impaired cognitive function in abstinent MA abusers (19), In addition, Chung et al. used WCST to indicate cognitive function (17). Salo et al. reported that MA abusers exhibited greater Stroop reaction time interference and group differences in the genu, but not in the splenium FA, through the Stroop attention test (24). As it is known, impulsivity can be broadly defined as behavioral actions without adequate forethought (25), and this has been considered as core impairments underpinning addictive behavior, which contribute to poor recovery and relapse (26). Some researches on other kinds of drugs, such as marijuana (27) and cocaine (28), have revealed that there are correlations between diffusion parameters and impulsivity behavior. However, there are few DTI studies that linked diffusion parameters to impulsive behavior in MA addicts. An investigator performed the correlations based on ROIs in the frontal areas of the brain, because previous studies have shown that impulsivity is mainly correlated to the frontal areas (23). In order to perform correlations in other brain regions and further determine the neural basis of impulsivity in MA dependence, diffusion parameters in the whole brain were correlated with the Barratt Impulsivity Scale (BIS) in the present study.

Therefore, a study on MA addicts was conducted and two hypotheses were obtained. First hypothesis: FA decreased in multiple brain regions, and MD, RD, and AD increased in the corresponding regions; Second hypothesis: BIS scores are much higher in the MA group, when compared to healthy controls (HCs), and the abnormalities of the diffusion parameters corresponded with the BIS scores in some tracts in the frontal areas of the brain.

## Materials and Methods

### Participants

The present study recruited 28 MA-dependent participants and 22 HCs. The participants in the MA group were collected from drug rehabilitation centers in Changsha, Zhuzhou and Yueyang (Hunan Province). All subjects were evaluated with a detailed drug use history and psychiatric evaluations during the face-to-face interview by trained staff of these drug rehabilitation centers. All participants provided a written informed consent. The experiment was approved by the ethics committee of the Second Xiangya hospital of Central South University. Inclusion criteria: positive urine test for MA and negative urine test for heroin, marijuana and ketamine; diagnosis of addiction based on the criteria outlined in the Fourth Edition of the Diagnostic and Statistical Manual of Mental Disorders (DSM-IV). Exclusion criteria: comorbid or history of psychiatric illness (e.g., schizophrenia and major depression unrelated to drug withdrawal), neurological disorder (e.g., multiple sclerosis, Parkinson's Disease, other primary degenerative brain diseases, and any brain infections or neoplasms), head trauma, or major chronic medical illnesses (e.g., diabetes, uncontrolled hypertension, and heart disease) that may confound the results or analysis of the present study; contraindications for magnetic resonance imaging (MRI) (e.g., metallic, electronic devices, or implants); pregnant subjects. Patients who met the aforementioned criteria underwent an MRI examination after 4–71 days of abstinence. HCs were recruited through WeChat, QQ, and other available media. These participants all fulfilled the same exclusion criteria as the MA users.

The background information was verified before the commencement of the brain imaging. The information gathered included the following demographics: age, gender, and educational attainment. Furthermore, the clinical characteristics were gathered include: MA use onset, duration of MA use, duration of addiction, and regular addiction dose per day. The data was lost in two MA addicts. Hence, merely the scores of 26 MA addicts were determined. The Fagerstrom Test for Nicotine Dependence (FTND) was also used to evaluate for cigarette smoking, and the Alcohol Use Disorder Identification Test (AUDIT) was used to evaluate for alcohol use. The demographic characteristics of the MA and control groups are presented in **Table 1**.

**Table 1:** Participant characteristics.

Demographic measures	MA	HC	t/χ^2^/Z	*p*
**Number**	28	22		
**Gender**	F: 4; M: 24	F: 3; M: 19	0.004	0.948
**Age^1^**	36.79 ± 8.561	40.91 ± 7.137	-1.816	0.076
**Education^2^**	9(8, 10.75)	9(9, 12)	-1.941	0.052
**FTND^2^**	5(4, 6)	4(2.75, 6.25)	-0.802	0.423
**AUDIT^2^**	1(0, 6.75)	3.5(0, 12)	-1.388	0.165
**MA use onset^3^**	28(15–43)			
**Duration of MA use(years) ^3^**	4.75 (1.5–12)			
**Duration of addiction(years)^3^**	3(0.5–11)			
**Regular addiction dose/day(g) ^3^**	0.5(0.03–2)			
**Abstinence(days) ^3^**	18(4–71)			
**BIS total score^1^**	86.82 ± 15.132	73.32 ± 16.889	2.865	0.006^*^
**BIS motor score^1^**	26.29 ± 5.076	22.32 ± 6.498	2.348	0.023^*^
**BIS attention score^1^**	30.96 ± 8.694	23.79 ± 8.456	2.807	0.007^*^
**BIS non-planning score^1^**	29.21 ± 6.094	27.21 ± 8.670	0.932	0.356

FTND, Fagerstrom Test for Nicotine Dependence; AUDIT, Alcohol Use Disorders Identification Test; BIS, Barratt Impulsivity Scale. The variables with the superscript 1 are expressed as mean ± standard deviation, the variables with the superscript 2 are represented by the median (P_25_, P_75_), the variables with the superscript 3 are represented by the median(range), and Data superscripted with asterisks(*) indicates significant differences between groups.

### MRI Acquisition

All MRI data were acquired on a 3T Siemens Skyra MRI scanner (Magnetom Skyra, Siemens, Germany) at the Second Xiangya hospital of Central South University, equipped with a 32-channel head coil. The MRI scanning included T1-weight imaging (T1WI), T2-weight imaging (T2WI), fluid-attenuated inversion recovery (FLAIR), three-dimensional magnetically prepared rapid acquisition gradient echo (3D MPRAGE) sequences, and diffusion tensor imaging (DTI). DTI was acquired with the following parameters: 64 diffusion directions at b=1,000 mm/s^2^ and b=0 mm/s^2^, TR=9,500 ms, TE=88 ms, 60 slices, slice thickness = 2 mm, slice spacing = 1 mm, FOV = 256 × 256 mm^2^, acquisition matrix = 256 × 256, and voxel size = 2 × 2 × 2 mm^3^. All subjects were placed in a supine position with foam padding between their head and the head coil to minimize head motion.

### Neuropsychological Test Acquisition

All participants completed the Barratt Impulsivity Scale version 11 (BIS-11). However, the data was lost in three HCs. Hence, merely the scores of 28 MA addicts and 19 HCs were determined. BIS-11 is a valid and reliable measure of impulsivity that factors in motor (10 items), attention (10 items), and non-planning (10 items) subscales (29). This has been wildly used around the world to measure one's impulsivity for fifty years. Each item is scored according to the frequency scale (1 refer to never; 4 refer to almost always/always).

### Image Analysis

All scans were processed using the PANDA software (30), which is a MATLAB toolbox that integrates FSL (https://fsl.fmrib.ox.ac.uk/fsl/fslwiki), Diffusion Toolkit (http://www.trackvis.org/dtk/), and MRIcron (https://www.nitrc.org/projects/mricron). PANDA can automatically process brain diffusion images through the following steps: (1) Image format conversion: The dcm2nii tool in MRIcron was used to convert the DICOM file into a Nifti image. (2) Brain extraction and estimate mask: The deletion of non-brain tissues and estimation of the brain mask. (3) Crop and eddy current/motion correction: The redundant parts of the images were cropped for memory reduction, and the images were corrected for the elimination of movements and eddy current-induced distortions. (4) DTI metric calculation: Diffusion tensor fitting and diffusion metrics, including FA, MD, AD, and RD, were calculated using the FSL DTIFit. (5) Spatial normalization: The diffusion metrics were spatially normalized to the Montreal Neurological Institute (MNI) space through FSL FNIRT. A TBSS analysis was performed on PANDA to exclude voxels in the gray matter and cerebrospinal fluid, and the mean FA skeleton image was thresholded with a default value of 0.2. The DTI metric images of all subjects in the WM skeleton were generated and computed in the statistical step.

### Statistical Analysis

The demographic characteristics and aspects of the behavioral data were analyzed using IBM SPSS Statistics 22.0 for Windows. Furthermore, unpaired two-sample *t*-test was performed for age. Chi-square tests were performed for gender. And Kruskal-Wallis test was performed for educational attainment, the FTND scores and AUDIT scores. These were used to determine whether the two groups significantly differed in terms of self-rating scores (FTND and AUDIT) and demographic characteristics. In addition, unpaired two-sample *t*-test was performed for the BIS-11 total and subscale scores. Next, TBSS analyses were performed using TBSS Statistics, which is a PANDA utility for non-parametric permutation inference on neuroimaging data. The matrix was designed on FSL, and a total of 5,000 permutations were performed for each contrast. The *P*-value images, which were fully corrected by threshold-free cluster enhancement (TFCE) (31), were thresholded at *P* < 0.05. Finally, the correlations between diffusion parameters and the BIS-11 total score, as well as the scores of the three subscales, were evaluated by partial correlation, using the FTND and AUDIT scores as covariates. A *P*-value of <0.05 was considered statistically significant.

## Results

The participant characteristic comparisons are presented in **Table 1**. The sample included 28 MA addicts and 22 HCs. All participants were righthanded and of Han Chinese descent. There were no significant between-group differences in age, gender, educational attainment, and FTND and AUDIT scores. The range of MA use onset is 15–43 years old; duration of MA use from 1.5 to 12 years; duration of MA addiction from 0.5 to 11 years; regular addiction dose is 0.03–2 grams.

**Figure 1:**
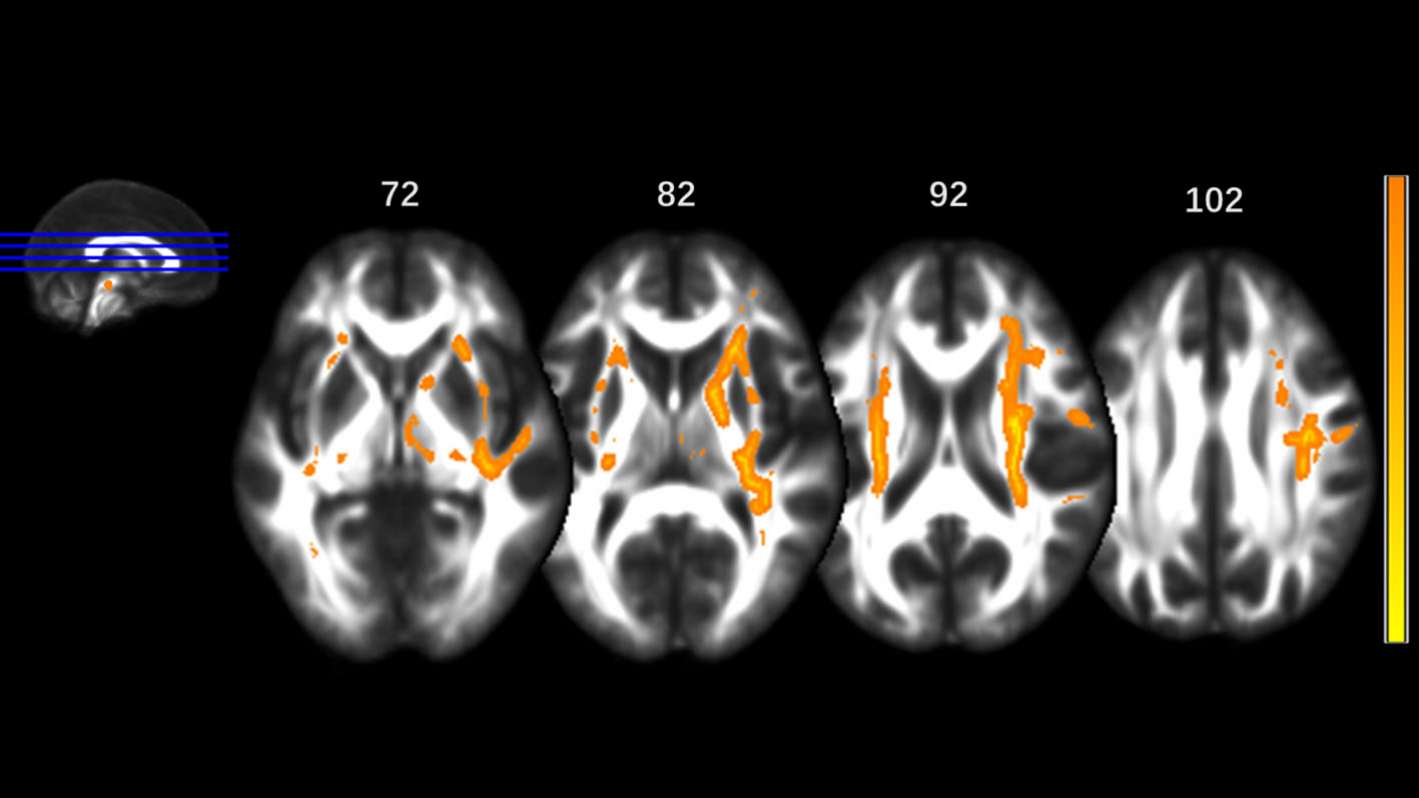
Significantly different tracts of lower fractional anisotropy (FA). These tracts are bilateral superior longitudinal fasciculus, sagittal stratum (including inferior fronto-occipital and inferior longitudinal fascicule), corona radiata, internal capsule, external capsule, genu of corpus callosum, fornix and stria terminalis, posterior thalamic radiation, cerebral peduncle, superior cerebellar peduncle, pontine crossing tract; tapetum, superior fronto-occipital fascicule in the right side and uncinate fasciculus in the left side. Orange represents FA higher value and yellow represents lower FA.

**Figure 2:**
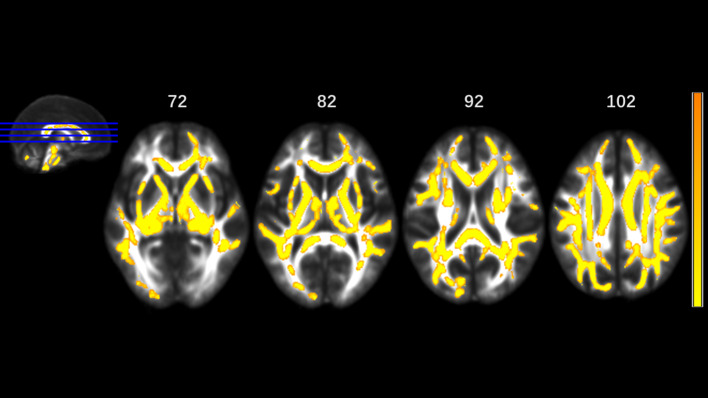
Significantly different tracts of higher axial diffusivity (AD). These tracts are bilateral superior longitudinal fasciculus, sagittal stratum (including inferior fronto-occipital and inferior longitudinal fascicule), corona radiata, internal capsule, external capsule, genu, body and splenium of corpus callosum, fornix and stria terminalis, posterior thalamic radiation, cerebral peduncle, superior cerebellar peduncle, pontine crossing tract, cingulum, corticospinal tract, medial lemniscus, and middle and inferior cerebellar peduncle; tapetum, superior fronto-occipital fascicule in the right side, and uncinate fasciculus in the left side. Orange represents lower AD value and yellow represents higher AD.

**Figure 3:**
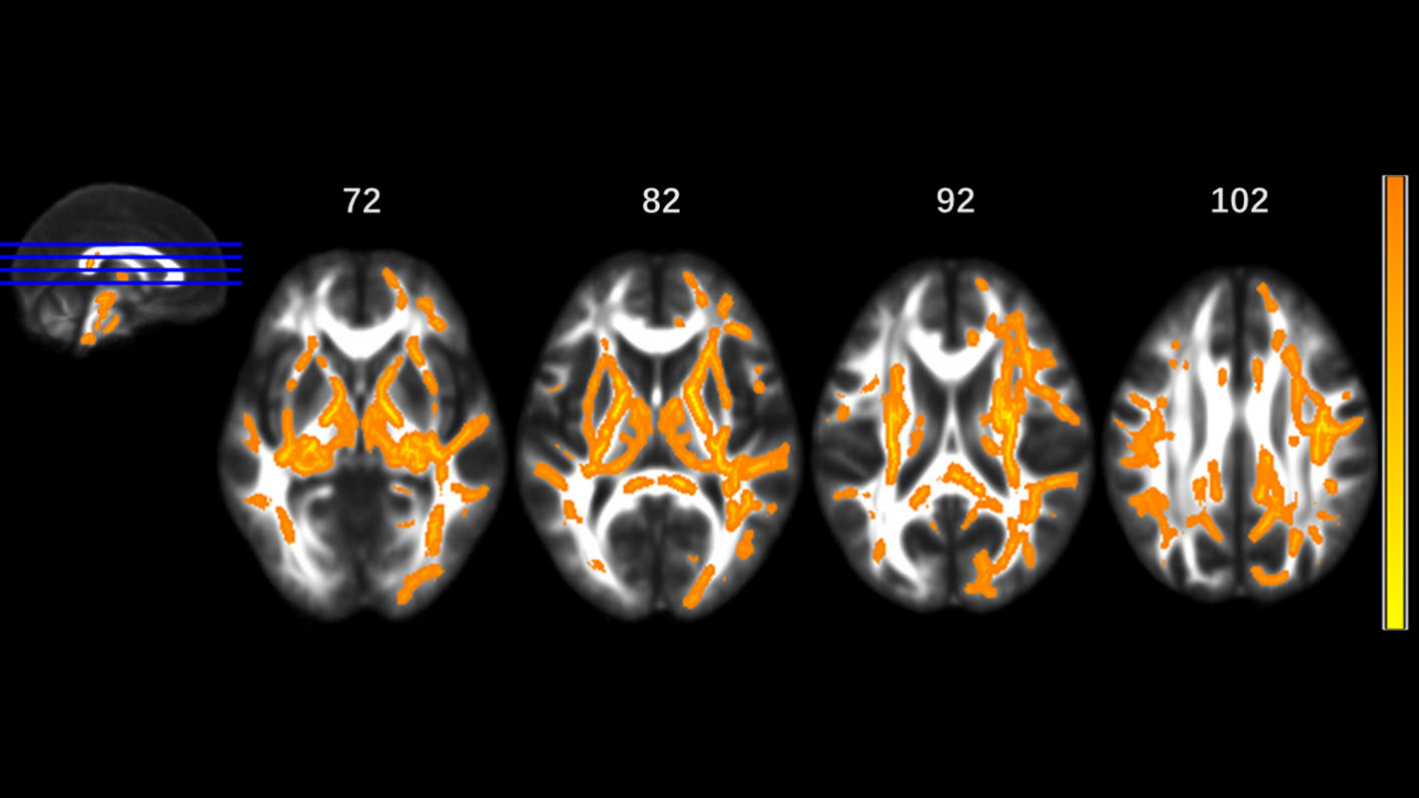
Significantly different tracts of higher radial diffusivity (RD). These tracts are bilateral superior longitudinal fasciculus, sagittal stratum (including inferior fronto-occipital and inferior longitudinal fascicule), corona radiata, internal capsule, external capsule, genu, body and splenium of corpus callosum, fornix and stria terminalis, posterior thalamic radiation, cerebral peduncle, superior cerebellar peduncle, pontine crossing tract, cingulum, corticospinal tract, medial lemniscus, and middle and inferior cerebellar peduncle; tapetum, superior fronto-occipital fascicule in the right side, and uncinate fasciculus in the left side. Orange represents lower RD value and yellow represents higher RD.

**Figure 4:**
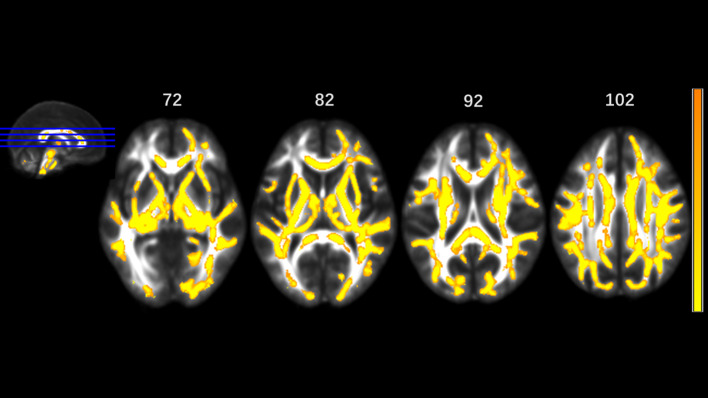
Significantly different tracts of higher mean diffusivity (MD). These tracts are bilateral superior longitudinal fasciculus, sagittal stratum (including inferior fronto-occipital and inferior longitudinal fascicule), corona radiata, internal capsule, external capsule, genu, body and splenium of corpus callosum, fornix and stria terminalis, posterior thalamic radiation, cerebral peduncle, superior cerebellar peduncle, pontine crossing tract, cingulum, corticospinal tract, medial lemniscus, and middle and inferior cerebellar peduncle.; tapetum, superior fronto-occipital fascicule in the right side, and uncinate fasciculus in the left side. Orange represents lower MD value and yellow represents higher MD.

MA addicts had significantly lower FA in a wide range of white matter, which included the bilateral corona radiata, internal capsule, superior longitudinal fasciculus, external capsule, inferior fronto-occipital fascicules, posterior thalamic radiation, sagittal stratum (including inferior longitudinal fasciculus and inferior fronto-occipital fascicules), fornix and stria terminalis, cerebral peduncle, superior cerebellar peduncle, genu of corpus callosum, and pontine crossing tract. Tapetum, superior fronto-occipital fascicule in the right side and uncinate fasciculus in the left side were compared to those in HCs. These tracts are presented in **Figure 1**. MA addicts also had higher AD, RD, and MD in the corresponding regions, when compared to HCs. In addition, the three parameters in the body, and the splenium of the corpus callosum, cingulum, corticospinal tract, medial lemniscus, and middle and inferior cerebellar peduncle were significantly higher in the MA group. These tracts are presented in **Figures 2**–**4**. Cluster details, including affected white matter tracts and voxel size are listed in **Table 2**.

**Table 2:** Cluster details with group difference in diffusion tensor imaging (DTI) measures.

Region name	Voxel size			
	FA	AD	RD	MD
Corona radiata R	6,265	11,658	12,359	15,109
Corona radiata L	1,695	9,635	5,345	8,937
Internal capsule R	5,106	6,071	7,300	7,331
Internal capsule L	1,717	6,683	6,361	6,777
Superior longitudinal fasciculus R	2,891	7,225	6,740	8,134
Superior longitudinal fasciculus L	4	6,047	4,301	6,694
External capsule R	2,090	2,482	3,025	3,173
External capsule L	1,360	2,418	3,105	3,004
Inferior fronto-occipital fasciculus R	910	1,378	1,671	1,697
Inferior fronto-occipital fasciculus L	608	722	1,740	1,546
Posterior thalamic radiation R	351	2,004	3,041	2,669
Posterior thalamic radiation L	11	3,004	1,023	2,228
Sagittal stratum (include inferior longitidinal fasciculus and inferior fronto-occipital fasciculus) R	303	1,091	1,330	1,338
Sagittal stratum (include inferior longitidinal fasciculus and inferior fronto-occipital fasciculus) L	47	576	905	822
Fornix/Stria terminalis R	219	967	932	977
Fornix/Stria terminalis L	6	956	938	984
Tapetum R	126	278	338	301
Tapetum L		419	5	180
Superior fronto-occipital fasciculus R	66	460	305	369
Superior fronto-occipital fasciculus L		183	86	171
Uncinate fasciculus L	34	169	272	266
Uncinate fasciculus R		246	251	255
Cerebral peduncle R	31	1,234	1,599	1,315
Cerebral peduncle L	6	1,764	1,715	1,816
Cerebellar peduncle R	17	1,448	882	1,389
Cerebellar peduncle L	1	1,447	733	1,136
Middle cerebellar peduncle		9,500	3,746	6,550
Corpus callosum	2	31,066	10,593	26,822
Pontine crossing tract	2	1,283	1,235	1,276
Corticospinal tract R		1,203	1,114	1,200
Corticospinal tract L		1,269	1,185	1,252
Cingulum R		1,018	1,827	2,536
Cingulum L		70	961	1,021
Medial lemniscus R		703	663	690
Medial lemniscus L		685	414	654

Affected white matter tracts and voxel size are listed. R, the right side; L, the left side. There are lower FA and higher AD, RD, MD in these voxels compared to healthy controls.

**Figure 5:**
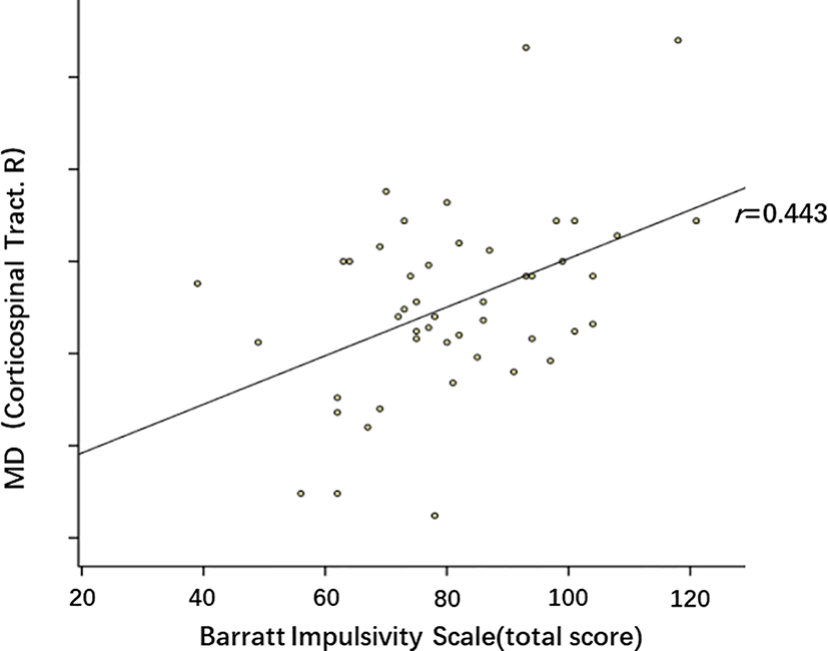
Partial correlation between mean diffusivity (MD) of right corticospinal tract and BIS-11 total scores. *p*=0.002, *r*=0.443. These dots roughly distribute as a positive correlation.

Subjects in the MA group had significantly higher BIS total scores (*P*=0.006), attention scores (*P*=0.007) and motor scores (*P*=0.023), when compared to the control group. After family-wise error (FWE) correction, the correlations between diffusion parameters and BIS-11 scores were as follows: No statistically significant relationships were detected between FA, AD, RD and BIS-11 scores. Higher MD in the right corticospinal tract was significantly associated with higher total scores (*r*=0.443, *P*=0.002). The partial correlation between MD of the right corticospinal tract and BIS-11 total scores is presented in **Figure 5**.

## Discussion

This DTI investigation of whole brain white matter integrity based on TBSS and its association with impulsivity revealed two main findings in MA group. (1) MA addicts had significantly lower FA and higher AD, RD, and MD in a wide range of white matter. (2) Subjects in the MA group had significantly higher BIS total score, attention and motor scores, when compared to the control group. The MD values in the corticospinal tract positively correlate with the BIS-11 total scores.

According to the first hypothesis of the investigators, MA addicts had significantly lower FA and higher AD, RD, and MD in a wide range of white matter. These tracts, in which the MA group was apparently different from HCs, were directly or indirectly connected in the present study (32, 33). Decreased FA and increased AD, RD, and MD demonstrate axonal damage. Many studies have confirmed this. A study on mice revealed that MA induces neurotoxicity associated with oxidative stress, microglial activation and pro-apoptotic changes (34). A MA user's case report revealed toxic leukoencephalopathy-like white matter rarefaction, loss of myelin, and axonal injury in the bilateral periventricular and centrum semiovale regions through MRI and autopsy (35). In addition, a review also illustrated that MA induces oxidative stress, neuroinflammation, and pro-apoptosis to dopaminergic neurotoxicity (36).

The results of other studies based on DTI were consistent with these present results (16–19). The present result included more tracts that were closer to the changes on the structure, metabolism, and function of a variety of brain regions reported by other studies (37). A study on children with prenatal MA exposure on TBSS revealed less brain regions than in the present findings. Furthermore, lower FA in the left external capsule, fornix and stria terminalis, higher MD and RD in corresponding regions, and higher AD in the left sagittal stratum were also found (38). Hence, there have discrepancies between the present study and that study, and the investigators suspect that MA has a more direct effect on the present subjects, and that the development and self-compensatory ability of the brains of children may be a possible reason. Similar to the present findings, a study on heroin dependent individuals displayed widespread FA reductions, which included clusters spanning the corpus callosum, thalamic radiation and parietal, frontal, and temporal tracts. However, that study reported lower AD, when compared to controls, in bilateral inferior frontal-occipital fasciculus, splenium of corpus callosum, and left inferior longitudinal fasciculus (39). The possible causes were the different drug types, and daily amounts and duration. Furthermore, the bidirectional change in AD both can represent neuronal damage. The potential explanation for the differences between the present study and the study conducted by Uhlmann et al. (23) is that part of the controls of their study were Cannabis users, and part of the MA addicts used more than one substance. In addition, the range of the duration of abstinence in the present study was smaller than the range of in their study, and these may cause of these differences.

Second main finding: Subjects in the MA group had significantly higher BIS total scores, motor and attention subscales scores, when compared to the control group. The MD values in the corticospinal tract positively correlate with the BIS-11 total scores. The higher level of impulsivity in the present study was in line with the studies conducted by Kogachi S et al. (40) and Uhlmann et al. (23). Our experiment shows that for MA addicts, motor and attention subscales are more sensitive than non-planning subscale, reflecting the increased impulsivity of MA addicts. As far as our concern, no other study on MA addicts has reached a similar result–subscales of different dimensions of BIS have different results. There is a study about marijuana found that marijuana smokers had significantly higher BIS total scores and motor subscale scores relative to HCs (27). The subscale headings (i.e., Motor, attention) may not describe the behaviors assessed by that subscale accurately. That is to say, each item of each subscale may represent the 3 dimensions of the BIS-11, so the results of our study eventually show that MA addicts are more impulse than HCs. The study conducted by Uhlmann A et al. revealed correlations in the frontal areas. For other substances, such as marijuana, the impulsivity is correlated with the left frontal FA values (27). However, a study on cocaine revealed a significant negative correlation between FA in the anterior corpus callosum and impulsivity behavior in cocaine-dependent subjects (28). One potential explanation for the differences between these studies and the present study was that more tracts were detected in the present study. Studies have shown that impulsivity is not a unitary construct, and that this may include the following: motor impulsivity, decision-making impulsivity, choice impulsivity, and reflection impulsivity (41). Furthermore, a meta-review reported that impulsivity comprises of a minimum of three neurocognitive components (26), such as response inhibition, reward discounting, and disadvantageous decision-making. There are several distinct neurocognitive mechanisms with separate neuroanatomical and neurochemical bases, and the different stimulant types and different subjects are the possible reasons. The study conducted by Lee B et al. revealed that the increase in BIS scores links to the decrease of striatal dopamine receptors in reduced methamphetamine dependence (42). The study conducted by Kohno et al. revealed that MA-dependent users exhibited a positive relationship for midbrain resting-state functional connectivity (RSFC) to the left ventral striatum with cognitive impulsivity. In addition, they also found that the ventral striatal's dopamine receptor availability negatively relates to RSFC between the midbrain and the striatum, orbitofrontal cortex, and insula in MA-dependent participants (43). These findings provide the physiology and functional link that may help explain the results in the present study. The study conducted by Winhusen et al. revealed that MA-dependents, relative to cocaine-dependent, participants evidenced significantly greater BIS-11 non-planning, and total scores (44). Hence, the correlation between different stimulant and diffusion parameters may be the direction of future research.

There were some limitations in the present study. First, the present study has a moderate sample size. Hence, more MA addicts and HCs should be involved in future studies to test and clarify the results in the present research. Second, the present study only has a self-report measure of impulsivity which is subjective relatively. Also, we excluded participants with current or past dependences on other drugs. But the truth of these answers is still unknown. Third, smoking and drinking may change the microstructure of the brain. Some studies have reported the effects of nicotine detected by DTI, and a study reported abnormal fronto-striatal tracts in young male tobacco smokers (45). In order to improve this deficiency and minimize the effects from nicotine and alcohol, the scores of FTND and AUDIT were matched, and a partial correlation was made using the FTND and AUDIT scores as covariates. Forth, the effects of MA abuse on the brain may be different between male and female MA abusers. In 2007, The study conducted by Chung A. et al. revealed lower FA values in frontal white matter, and more errors in the Wisconsin Card Sorting test were found only in male MA abusers (17). Salo et al. reported that male MA abusers exhibited greater ADC and λ1 eigenvalues within the splenium of the corpus callosum (24). Further studies should be performed to determine whether different genders have different impairments, and detect influencing factors, such as oestrogen, and other hormones, which should be taken into account. Finally, a longitudinal study should be performed to determine the changes in the long-term stages.

## Data Availability Statement

The original contributions presented in the study are included in the article/supplementary materials; further inquiries can be directed to the corresponding author.

## Ethics Statement

The studies involving human participants were reviewed and approved by the ethics committee of the Second Xiangya hospital of Central South University. The patients/participants provided their written informed consent to participate in this study.

## Author Contributions

SH designed the study. SH and CY conducted behavioral and imaging analyses. SH, WY, and JiL conducted the assessments. JuL modiﬁed the manuscript and supervised the study. SH wrote the ﬁrst draft and all authors provided input to the ﬁnal version of the manuscript.

## Funding

This study was funded by the National Natural Science Foundation of China (81671671, 61971451), the Natural Science Foundation of Hunan Province, China (2015JJ4081), the National Key Research Development Program of China (2016YFC0800908) and the key R & D projects in Hunan Province (2019SK2131).

## Conflict of Interest

The authors declare that the research was conducted in the absence of any commercial or financial relationships that could be construed as a potential conflict of interest.

The handling editor declared a shared affiliation, though no other collaboration, with several of the authors, WY, JiL, CY, JuL.
